# Genetically proxied lean mass and risk of Alzheimer’s disease: mendelian randomisation study

**DOI:** 10.1136/bmjmed-2022-000354

**Published:** 2023-06-29

**Authors:** Iyas Daghlas, Malik Nassan, Dipender Gill

**Affiliations:** 1 Department of Neurology, University of California San Francisco, San Francisco, California, USA; 2 Mesulam Center for Cognitive Neurology and Alzheimer’s Disease, Northwestern University, Chicago, Illinois, USA; 3 Department of Epidemiology and Biostatistics, Imperial College London, London, UK; 4 Chief Scientific Advisor Office, Research and Early Development, Novo Nordisk, Copenhagen, Denmark

**Keywords:** Genetics, Dementia, Neurology, Epidemiology

## Abstract

**Objective:**

To examine whether genetically proxied lean mass is associated with risk of Alzheimer’s disease.

**Design:**

Mendelian randomisation study.

**Setting:**

The UK Biobank study and genome wide association study meta-analyses of Alzheimer’s disease and cognitive performance.

**Participants:**

Summary level genetic data from: 450 243 UK Biobank participants with impedance measures of lean mass and fat mass; an independent sample of 21 982 patients with Alzheimer’s disease and 41 944 controls without Alzheimer’s disease; a replication sample of 7329 patients with Alzheimer’s disease and 252 879 controls; and 269 867 individuals taking part in a genome wide association study of cognitive performance.

**Main outcome measure:**

Effect of genetically proxied lean mass on the risk of Alzheimer’s disease, and the related phenotype of cognitive performance.

**Results:**

An increase in genetically proxied appendicular lean mass of one standard deviation was associated with a 12% reduced risk of Alzheimer’s disease (odds ratio 0.88, 95% confidence interval 0.82 to 0.95, P=0.001). This finding was replicated in an independent cohort of patients with Alzheimer’s disease (0.91, 0.83 to 0.99, P=0.02) and was consistent in sensitivity analyses that are more robust to the inclusion of pleiotropic variants. Higher genetically proxied appendicular lean mass was also associated with increased cognitive performance (standard deviation increase in cognitive performance for each standard deviation increase in appendicular lean mass 0.09, 95% confidence interval 0.06 to 0.11, P=0.001), and adjusting for potential mediation through genetically proxied cognitive performance did not reduce the association between appendicular lean mass and risk of Alzheimer’s disease. Similar results were found for the outcomes of Alzheimer’s disease and cognitive performance when the risk factors of genetically proxied trunk lean mass and whole body lean mass were used, respectively, adjusted for genetically proxied fat mass.

**Conclusions:**

These findings suggest that lean mass might be a possible modifiable protective factor for Alzheimer’s disease. The mechanisms underlying this finding, as well as the clinical and public health implications, warrant further investigation.

**WHAT IS ALREADY KNOWN ON THIS TOPIC**

- Although numerous studies have linked obesity to the risk of Alzheimer’s disease, high quality data exploring associations between lean muscle mass and risk of Alzheimer’s disease are lacking

**WHAT THIS STUDY ADDS**

- Based on naturally randomised human genetics data, individuals randomised to lifelong higher lean muscle mass had a 12% lower risk of Alzheimer’s disease and scored higher for cognitive performance

**HOW THIS STUDY MIGHT AFFECT RESEARCH, PRACTICE, OR POLICY**

- These findings suggest that lean mass might be a protective factor for Alzheimer’s disease
- Further research is warranted to investigate the clinical and public health implications of these findings

## Introduction

Despite the steady increase in the prevalence of Alzheimer’s disease,^1^ no effective treatments for this devastating disease exist. Prevention of Alzheimer’s disease through identification of modifiable risk factors is thus a key public health aim. A systematic review of observational studies of Alzheimer’s disease estimated that a third of patients with incident Alzheimer’s disease were attributable to modifiable risk factors, including mid-life obesity.^2^ Putative mediators for the association between obesity and Alzheimer’s disease include increased inflammation, insulin resistance, and increased levels of amyloid β in adipose tissue.^3^

In addition to obesity, several different measures of body composition have been studied for their association with Alzheimer’s disease. Lean mass, a proxy for muscle mass defined as the difference between total mass and fat mass, has consistently been found to be reduced in patients with Alzheimer’s disease compared with carefully selected controls.^4–6^ The interpretation of this finding has been that the reduction in lean mass is a result of Alzheimer’s disease, but most studies used case-control study designs which cannot determine the direction of effect. These findings could reflect a causal effect of higher lean mass on reduced risk of Alzheimer’s disease. Such an effect might partially explain the protective association between physical activity and exercise and reduced risk of Alzheimer’s disease in observational studies.^7 8^ Moreover, associations from observational studies might be biased by residual confounding or reverse causality and therefore not causal.

Naturally randomised data may be used as an alternative to conventional observational studies to investigate causal relations between risk factors and diseases. The mendelian randomisation paradigm uses germline genetic variants as instrumental variables to proxy the effect of modifying a risk factor on a disease outcome. Germline genetic variants are randomly allocated at gametogenesis and their use as proxies for a specific risk factor is therefore less confounded by environmental variables. Germline genetic variants are fixed at gametogenesis and do not change during the life course, and so their use as proxies for a specific risk factor is not biased by reverse causality. This effect is important in Alzheimer’s disease, because the preclinical disease process might influence body composition.^9^

The aim of this study was to use mendelian randomisation to investigate the effect of genetically proxied lean mass on the risk of Alzheimer’s disease and the related phenotype of cognitive performance, which is causally related to the risk of Alzheimer’s disease.^10^ We hypothesised that genetically proxied higher lean mass would be associated with a reduced risk of Alzheimer’s disease and with higher cognitive performance.

## Methods

### Genetic associations with lean mass

As genetic proxies for lean mass, we selected single nucleotide polymorphisms (SNPs, used interchangeably with genetic variants) that were associated in a genome wide association study (GWAS) with appendicular lean mass at genome wide significance (P<5×10^-8^).^11^ This GWAS used phenotypic and genetic data from 450 243 participants in the UK Biobank cohort (mean age 57 years).^11 12^ Appendicular lean mass more accurately reflects the effects of lean mass than whole body lean mass, which includes smooth and cardiac muscle.^11^ Fat free mass (used interchangeably with lean mass) was determined with bioimpedance, measured by the Tanita BC418MA body composition analyser, with a standardised UK Biobank protocol.^12^

Appendicular lean mass was calculated as the sum of lean mass in the arms and legs. Appendicular lean mass was residualised for age, age^2^, UK Biobank genotyping array, top 10 principal components of ancestry, UK Biobank assessment centre, and appendicular fat mass (the sum of fat mass in all four extremities). Adjustment for the principal components of ancestry minimises confounding of genetic associations by ancestry, which is a key assumption of mendelian randomisation. These residuals were normalised into normalised quantiles of a standard normal distribution and therefore the effects of SNPs are reported in standard deviation units. The measure of appendicular lean mass derived from bioimpedance was validated in a subset of UK Biobank individuals with measurements of body composition by dual energy x ray absorptiometry (DEXA).^11^ Moreover, the association between the variants and appendicular lean mass was consistent across all age groups included in the analysis (38-45, 46-50, 51-55, 56-60, 61-65, and 66-74 years).^11^

In secondary analyses, we used genetic variants associated with trunk lean mass (n=447 990) and whole body lean mass (n=448 322) in GWAS performed in the UK Biobank. These GWAS were adjusted for age, age^2^, sex, age×sex, age^2^×sex, and the top 20 principal components of ancestry. In these analyses, we also performed multivariable mendelian randomisation analyses adjusting, respectively, for trunk fat mass (n=448 068) and whole body fat mass (n=447 626). All SNP effects are in standard deviation units.

### Genetic associations with Alzheimer’s disease, cognitive performance, and hippocampal volume

Recent GWAS of Alzheimer’s disease^13^ included proxy patients that were identified based on a family history of dementia, and so were likely to include patients with non-Alzheimer’s disease dementia. To ensure the relevance of our results to Alzheimer’s disease, we restricted analysis to the primary analytic outcome of clinically diagnosed late onset Alzheimer’s disease. We obtained genetic associations with Alzheimer’s disease from the first stage of the 2019 International Genomics of Alzheimer’s Project meta-analysis of GWAS of Alzheimer’s disease.^14^ All participants were of European ancestry. Genetic associations were adjusted for age, sex, and principal components of ancestry.

For replication, we obtained genetic associations with Alzheimer’s disease from release six of the FinnGen consortium.^15^ FinnGen is a Finnish cohort that combines health record data with genomic data. We used the curated G6_AD_WIDE phenotype, which combines the ICD-10 (international classification of diseases, 10th revision) codes (F00 and G30) and ICD-9 (international classification of diseases, ninth revision) codes (3310) with the purchase history of drug treatments for Alzheimer’s disease (including donepezil, memantine, and rivastigmine). These drug treatments are not exclusively prescribed to patients with Alzheimer’s disease, and so we anticipated that some patients were misclassified. ICD codes were obtained from inpatient and outpatient settings. No sample overlap was present between the risk factor sample (UK Biobank) and either of these cohorts of Alzheimer’s disease.

Genetic associations with cognitive tasks were obtained from the largest available meta-analysis of 14 GWAS of cognitive performance in individuals of European ancestry (n=269 867).^16^ Each cohort extracted a score representing a common latent g factor (or general intelligence) that contributes to multiple dimensions of cognition.^17 18^ Genetic associations with cognitive tasks are reported in standard deviation units. Genetic associations with hippocampal volume were obtained from the largest publicly available GWAS meta-analysis of hippocampal volume, defined by magnetic resonance imaging, that did not overlap with the UK Biobank (n=30 717, all individuals of European ancestry).^19^ Genetic associations were adjusted for intracranial volume (reported as mm^3^).

### Selection of genetic proxies for lean mass

The mendelian randomisation method requires that genetic variants used as proxies are strongly associated with the risk factor of interest and are not in high linkage disequilibrium with one another. To select independent variants meeting these criteria, we first identified the set of genome wide significant (P<0.001) variants overlapping between the risk factor GWAS (eg, lean mass) and the respective outcome GWAS (eg, Alzheimer’s disease). We then clumped the variants within a 10 Mb window using a between-SNP r^2^<0.001 (the r^2^ value is a measure of the tendency for genetic variants to be inherited together; calculated using the 1000G European reference panel).^20^ We calculated the variance explained by the variants used to proxy appendicular lean mass.^21^ We also checked the appendicular lean mass variant list to ensure that no genetic proxies were present within the *APOE* gene region (Chr19:45,116,911-46,318,605).^14^ This variant selection process was used for all analyses.

### Statistical analysis

Genetic associations between lean mass and Alzheimer’s disease were harmonised by aligning beta coefficients to the same effect allele.^20^ We used the random effects inverse variance weighted method as the primary mendelian randomisation approach. This method regresses the SNP-outcome association on the SNP-risk-factor association and weights the regression by the inverse of the standard error of the SNP-outcome association. The estimand is the effect of a standard deviation increase in lean mass on the risk of Alzheimer’s disease (or on standard deviation units of cognitive performance). All mendelian randomisation analyses were performed with the TwoSampleMR version 4.2.1.^20^ Online supplemental figure 1 shows a causal diagram illustrating the hypotheses for our analyses.

10.1136/bmjmed-2022-000354.supp1 Supplementary data

We performed several sensitivity analyses. The causal effect estimated in a mendelian randomisation analysis is unbiased if the effect of the genetic proxies on Alzheimer’s disease is mediated through lean mass and not through other causal pathways. To test this assumption of no horizontal pleiotropy, we performed sensitivity analyses that are more robust to the inclusion of pleiotropic variants including the following methods: weighted median,^22^ mendelian randomisation Egger,^23^ and penalised weighted median.^24^

To test for associations in the reverse direction, we performed mendelian randomisation analyses to test for the association between genetically proxied liability to Alzheimer’s disease^14^ and appendicular lean mass. The appendicular lean mass GWAS adjusted for appendicular fat mass, and the use of GWAS adjusted for heritable covariates that might introduce collider bias in a mendelian randomisation analysis.^25^ This bias can be overcome with multivariable mendelian randomisation rather than genetic associations adjusted for covariates.^26^ We therefore repeated the analyses with genetically predicted lean mass and trunk mass adjusted, respectively, for genetically predicted lean fat mass and trunk fat mass in multivariable mendelian randomisation analyses.^27^ This multivariable mendelian randomisation approach also allows estimation of the association between genetically proxied fat mass and Alzheimer’s disease adjusted for lean mass.

Genetic variants for use in multivariable mendelian randomisation were identified by pooling all genome wide significant SNPs for both risk factors, ordering by lowest P value, and clumping as described above. We used the regression based multivariable mendelian randomisation method which regresses the SNP-outcome associations on the two SNP-risk-factor associations, with the intercept fixed at zero with a random effects model.^20^ We also performed multivariable mendelian randomisation analyses adjusting for genetically proxied height with a dataset that did not overlap with the UK Biobank (n=253 288 participants of European ancestry).^28^ Finally, we compared associations between genetically proxied lean mass and Alzheimer’s disease with conventional epidemiological proxies for adiposity, such as body mass index (n=806 834 participants of European ancestry, 451 SNPs) and waist-hip ratio adjusted for body mass index (WHRadjBMI; n=694 649 participants of European ancestry, 258 SNPs).^29^

To examine whether the effect of lean mass on cognitive performance is mediated through Alzheimer’s disease, we performed multivariable mendelian randomisation analyses adjusting the lean mass effect for liability to Alzheimer’s disease. To examine whether the effect of lean mass on Alzheimer’s disease is mediated through cognitive performance, we performed multivariable mendelian randomisation analyses controlling the lean mass association for cognitive performance. Finally, in post hoc analyses, we tested whether coronary artery disease (132 variants^30^) could function as a putative mediator for the association between appendicular lean mass and risk of Alzheimer’s disease by first examining its association with Alzheimer’s disease. If this association was significant, we planned to perform multivariable mendelian randomisation analyses adjusting the association between appendicular lean mass and Alzheimer’s disease for effects of the proxies on the risk of coronary artery disease.

### Patient and public involvement

Patients or members of the public were not involved in the design of the study, interpretation of the results, or drafting of the manuscript. We currently have no plans to share the results with research participants or public communities. Results will be disseminated through a press release.

## Results

**Figure 1:**
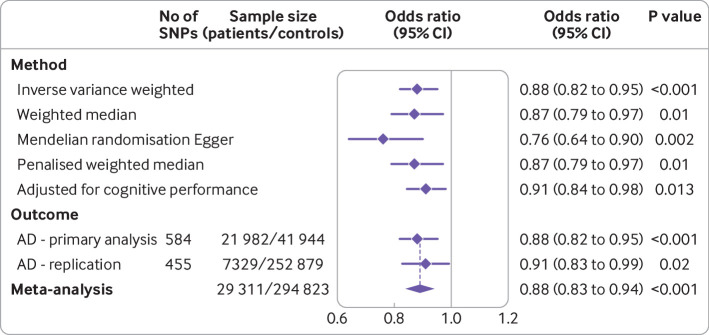
Forest plot of mendelian randomisation estimates, sensitivity analyses, and replication analysis for the estimated effect of appendicular lean mass on risk of Alzheimer’s disease. Primary mendelian randomisation analysis based on the Alzheimer’s genome wide association study meta-analysis (21 982 patients and 41 944 controls). Point estimates (diamonds) are expressed as the odds ratio of Alzheimer’s disease for each standard deviation increase in appendicular lean mass (with 95% confidence intervals). Genetic variants (n=584) were used as proxies for appendicular lean mass adjusted for appendicular fat mass. Multivariable mendelian randomisation adjusting for genetically proxied cognitive performance (total 608 variants) was used to estimate the adjusted for cognitive performance effect. Also shown is replication and fixed effects meta-analysis based on genome wide association data for Alzheimer’s disease from the FinnGen consortium. AD=Alzheimer’s disease; CI=confidence interval; SNPs=single nucleotide polymorphisms

We identified 584 variants the meeting criteria for use as genetic proxies for lean mass (online supplemental table 1). None of these variants was located within the *APOE* gene region. The variants in aggregate explained 10.3% of the variance in appendicular lean mass. A standard deviation increase in genetically proxied lean mass was associated with a 12% reduced risk of Alzheimer’s disease (odds ratio 0.88, 95% confidence interval 0.82 to 0.95, P<0.001; figure 1 and online supplemental figure 2). This finding was replicated in the independent FinnGen consortium (0.91, 0.83 to 0.99, P=0.02; figure 1).

10.1136/bmjmed-2022-000354.supp2 Supplementary data

10.1136/bmjmed-2022-000354.supp3 Supplementary data

**Figure 2:**
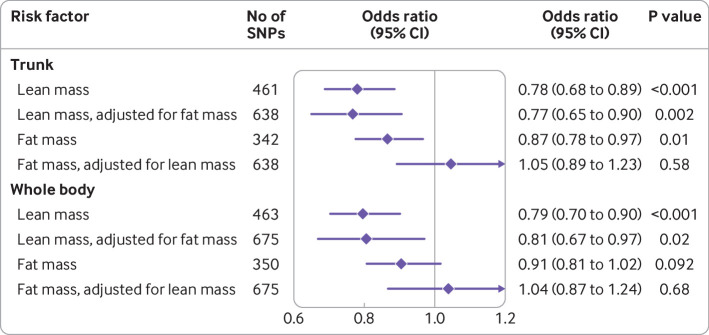
Forest plot of univariable and multivariable mendelian randomisation estimates for the estimated effect of trunk and whole body impedance measures on risk of Alzheimer’s disease (21 982 patients and 41 944 controls). Point estimates (diamonds) are expressed as the odds ratio of Alzheimer’s disease for each standard deviation increase in the respective risk factor (with 95% confidence intervals). The univariable and multivariable random effects inverse variance weighted mendelian randomisation methods were used to calculate effect estimates. CI=confidence interval; SNPs=single nucleotide polymorphisms

Results were similar in sensitivity analyses with models more robust to inclusion of pleiotropic variants (figure 1). Higher genetically proxied trunk and whole body lean mass also had protective associations with the risk of Alzheimer’s disease (figure 2). These estimates were not markedly changed with adjustment for genetically proxied fat mass. In univariable mendelian randomisation analyses, genetically proxied fat mass had a protective effect on the risk of Alzheimer’s disease (figure 2). Adjusting for genetic associations of these genetic variants with lean mass reduced these effects to the null, however, suggesting confounding by pleiotropic effects of fat mass proxies on lean mass (figure 2).

Adjusting for genetic associations with height gave similar point estimates across both the UK Biobank and FinnGen cohorts, and meta-analysis across both cohorts gave a significant association (0.90, 95% confidence interval 0.80 to 0.997, P=0.04; online supplemental table 3). Genetic liability to Alzheimer’s disease was associated with higher appendicular lean mass (0.01, 0.001 to 0.02, P=0.03; online supplemental table 2). In contrast with the association between appendicular lean mass and Alzheimer’s disease, we found no evidence for an association between body mass index (0.81, 0.62 to 1.07, P=0.13) or WHRadjBMI (0.80, 0.40 to 1.20, P=0.28) and risk of Alzheimer’s disease.

10.1136/bmjmed-2022-000354.supp4 Supplementary data

10.1136/bmjmed-2022-000354.supp5 Supplementary data

**Figure 3:**
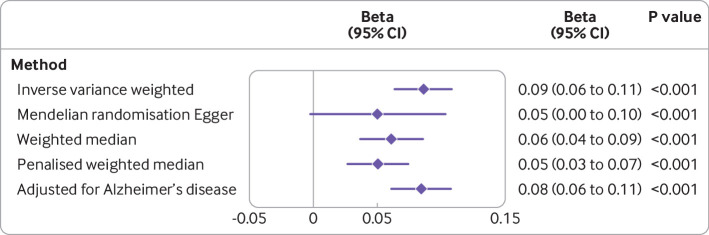
Forest plot of mendelian randomisation estimates for the estimated effect of appendicular lean mass, adjusted for appendicular fat mass, on cognitive performance. Point estimates (beta coefficients, diamonds) are expressed as the standard deviation change in cognitive performance for each standard deviation increase in appendicular lean mass (with 95% confidence intervals). Genetic variants (n=601) were used as proxies for appendicular lean mass adjusted for appendicular fat mass. Multivariable mendelian randomisation adjusting for genetic liability to Alzheimer’s disease (total 578 genetic variants) was used to estimate the adjusted for Alzheimer’s disease effect. CI=confidence interval

**Figure 4:**
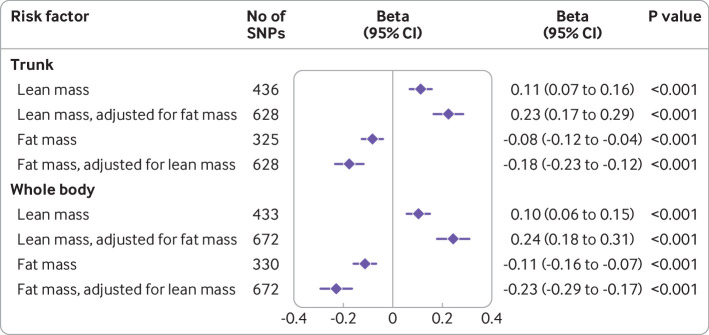
Forest plot of univariable and multivariable mendelian randomisation estimates for the estimated effect of trunk and whole body impedance measures on cognitive performance. Point estimates (beta coefficients, diamonds) are expressed as the standard deviation change in cognitive performance for each standard deviation increase in the risk factor (with 95% confidence intervals). The univariable and multivariable random effects inverse variance weighted method was used to calculate effect estimates. CI=confidence interval; SNPs=single nucleotide polymorphisms

Genetically proxied appendicular lean mass was positively associated with cognitive performance (0.09 standard deviation unit increase in cognitive performance for each standard deviation increase in appendicular lean mass, 0.06 to 0.11, P<0.001; figure 3 and online supplemental figure 3). This effect was consistent across the sensitivity analyses (figure 3) but adjustment for genetically proxied cognitive performance in multivariable mendelian randomisation analyses did not reduce the association between genetically proxied lean mass and Alzheimer’s disease (figure 1). Adjustment for genetic liability to Alzheimer’s disease did not influence the effect of appendicular lean mass on cognitive performance. Higher genetically proxied trunk and whole body lean mass were also associated with higher cognitive performance (figure 4). Adjustment for fat mass increased the magnitude of the mendelian randomisation estimate, a pattern attributable to the inverse association between genetically proxied fat mass and cognitive performance (figure 4). In contrast, the association between genetically proxied fat mass and lower cognitive performance was increased in magnitude on adjustment for genetically proxied lean mass, suggesting that pleiotropic effects might have masked a true detrimental effect of fat mass on cognitive performance (figure 4).

10.1136/bmjmed-2022-000354.supp6 Supplementary data

We found no evidence for an association between genetically proxied appendicular lean mass and hippocampal volume (3.2 mm^3^, 95% confidence interval −18 to 25, P=0.78). We also looked at whether coronary artery disease might function as a mediator for the effects of appendicular lean mass on the risk of Alzheimer’s disease. The univariable mendelian randomisation association of genetic liability to coronary artery disease on the risk of Alzheimer’s disease was null, however, and so we did not perform multivariable analyses (odds ratio of Alzheimer’s disease for each log odds increase in liability of coronary artery disease 1.01, 0.91 to 1.10, P=0.91).

## Discussion

### Principal findings

In this mendelian randomisation analysis, we found evidence supporting a protective effect of genetically proxied lean mass on the risk of Alzheimer’s disease. This finding was replicated in an independent cohort and was consistent across multiple sensitivity analyses and different measures of lean mass. Genetically proxied lean mass was associated with increased cognitive performance, but this association did not explain the protective effect of lean mass on the risk of Alzheimer’s disease. Genetically proxied body fat adjusted for lean mass was not associated with the risk of Alzheimer’s disease but was associated with reduced cognitive performance.

These analyses provide new evidence supporting a cause-and-effect relation between lean mass and risk of Alzheimer’s disease. These results are consistent with previous observational analyses showing an inverse relation between lean mass and Alzheimer’s disease^4 5 31^ and with a recent mendelian randomisation analysis.^32^ Our findings refute a large effect of fat mass on the risk of Alzheimer’s disease and highlight the importance of distinguishing between lean mass and fat mass when investigating the effect of adiposity measures on health outcomes. This null association between fat mass and risk of Alzheimer’s disease is in line with our analyses and previously published analyses which have not shown an effect of body mass index or of waist-hip ratio on the risk of Alzheimer’s disease.^33 34^ This finding could be both a result of a combined effect of these phenotypes being imperfect proxies for adiposity and an overall minimal effect of adiposity on the risk of Alzheimer’s disease.

Our findings need to be replicated with independent lines of complementary evidence before informing public health or clinical practice. Also, more work is needed to determine the cut-off values for age and degree of pathology of Alzheimer’s disease after which modifications of lean mass might no longer reduce the risk of Alzheimer’s disease. Should these findings be supported in future studies, public health efforts to shift the population distribution of lean mass, potentially through campaigns to promote exercise and physical activity, might reduce the population burden of Alzheimer’s disease.^35^

### Mechanistic insights of this study

Our results suggest that appendicular lean mass does not exert its effect on Alzheimer’s disease through volumetric changes in the hippocampus, but results from these analyses were imprecise. Furthermore, hippocampal volume is generally considered to be a consequence rather than a cause of Alzheimer’s disease and is not uniformly reduced in volume across all subtypes of Alzheimer’s disease.^36^ Also, results from our primary analysis were further corroborated by the finding that genetically proxied lean mass was associated with increased cognitive performance. This finding is in keeping with previous observational studies and reviews investigating the relation between sarcopenia and impaired cognition.^37–39^ Considering the positive association with cognitive performance, we considered whether part of the effect of lean mass on the risk of Alzheimer’s disease might be attributed to increased cognitive reserve.^40^ The point estimate for the effect of appendicular lean mass on Alzheimer’s disease was similar after adjusting for cognitive performance in multivariable mendelian randomisation, however, suggesting minimal mediation through this pathway.

Several mendelian randomisation analyses have identified the effects of cardiometabolic risk factors on the risk of Alzheimer’s disease, which could be further investigated as potential mediators of the effect of lean mass on Alzheimer’s disease in future analyses. Alternative potential pathways include insulin resistance^41^ and blood pressure^42^ (although with mixed results^43 44^). However, our analysis with coronary artery disease as a risk factor suggests that the burden of atherosclerotic disease is unlikely to be a relevant mediator.

Finally, the effect of lean mass on Alzheimer’s disease and cognitive performance could also be mediated by new mechanisms, including by muscle secretion of circulating centrally acting myokines.^45 46^ Potentially relevant secreted myokines include irisin, brain derived neurotrophic factor 5, and cathepsin B. Identification of the key causal pathways might lead to development of treatments that harness and potentiate the neuroprotective effects of lean mass.

### Strengths and limitations of this study

Our analysis had several strengths. Firstly, the mendelian randomisation approach is less susceptible to bias from residual confounding and reverse causality. Secondly, we replicated the effect of genetically proxied lean mass on Alzheimer’s disease in an independent population and with independent measures of lean mass. Thirdly, we accounted for the independent effects of lean mass and fat mass on the risk of Alzheimer’s disease. Finally, we found that collider bias was unlikely to influence our results because of the null effect of fat mass on the risk of Alzheimer’s disease, and the consistency of findings in our sensitivity analyses.

Our analysis had several limitations. Firstly, the bioimpedance measures only predict, but do not directly measure, lean mass. However, these genetic proxies were robustly associated with lean mass, measured by DEXA, in a subpopulation of the UK Biobank.^11^ Secondly, despite consistency across several sensitivity analyses, our results could be biased by horizontal pleiotropy.^31^ Thirdly, this mendelian randomisation approach only estimated the effect of linear changes around the population mean of lean mass and cannot be extrapolated to infer the consequences of extremes lean mass (eg, severe sarcopenia). Fourthly, our approach did not look at whether a critical window of risk factor timing exists during which lean mass has a role in influencing the risk of Alzheimer’s disease and after which interventions would no longer be effective. Similarly, we cannot determine whether increasing lean mass could reverse the pathology of Alzheimer’s disease in patients with preclinical disease or minor cognitive impairment. Answering these questions with mendelian randomisation would require the use of different sets of genetic variants proxying lean mass at different points in the life course and would be worthy topics for future analysis.

Future work should also investigate whether the effects of appendicular lean mass are applicable to other subtypes of dementia, such as vascular and mixed dementia. The cohorts used to define patients with Alzheimer’s disease did not use pathological definitions of Alzheimer’s disease and so could have included patients with mixed or vascular dementia. If an association exists between lean mass and vascular dementia but not with Alzheimer’s disease, then this finding might be expected to bias our results away from the null. In addition, our analyses were performed in relatively healthy populations of European ancestry which might affect their generalisability, particularly to other ancestry groups. Furthermore, the cohorts contributing to the cognitive performance GWAS likely had some degree of phenotypic heterogeneity because of the differences in cognitive tests and the settings in which they were given. However, g factors extracted from different tests correlated highly (r up to 1.0).^17 18^

Finally, we found an association between genetic liability to Alzheimer’s disease and higher appendicular lean mass. Although this finding might be consistent with some degree of reverse causality, we caution against this interpretation. This association was nominally significant and represented a small effect size. The magnitude and statistical strength of the association between appendicular lean mass and Alzheimer’s disease was much stronger. Also, we had no independent dataset to replicate this finding. We also highlight that the opposite direction of effect would be expected if a shared aetiology, rather than a causal effect, between appendicular lean mass and Alzheimer’s disease was explaining our findings for Alzheimer’s disease.

### Conclusions

In this study, we identified genetic support for a protective effect of lean mass on the risk of Alzheimer’s disease and on higher cognitive performance. Further investigation is warranted to understand the clinical and public health implications of these findings.

## Data Availability

Data are available in a public, open access repository. Data are available upon reasonable request. All data supporting these analyses are publicly available. The code used for mendelian randomisation analyses will be uploaded to the following folder on publication of the manuscript: https://drive.google.com/drive/folders/14cYM-CCddSNX6NURpVoc7pHKdm4xJ9N8?usp=share_link.
