# Supplementary figures and images for: Genetically proxied lean mass and risk of Alzheimer’s disease: mendelian randomisation study
Source: BMJ Med. 2023 Jun 29;2(1):e000354. doi: 10.1136/bmjmed-2022-000354 (PMC10410880; doi:10.1136/bmjmed-2022-000354)

A.

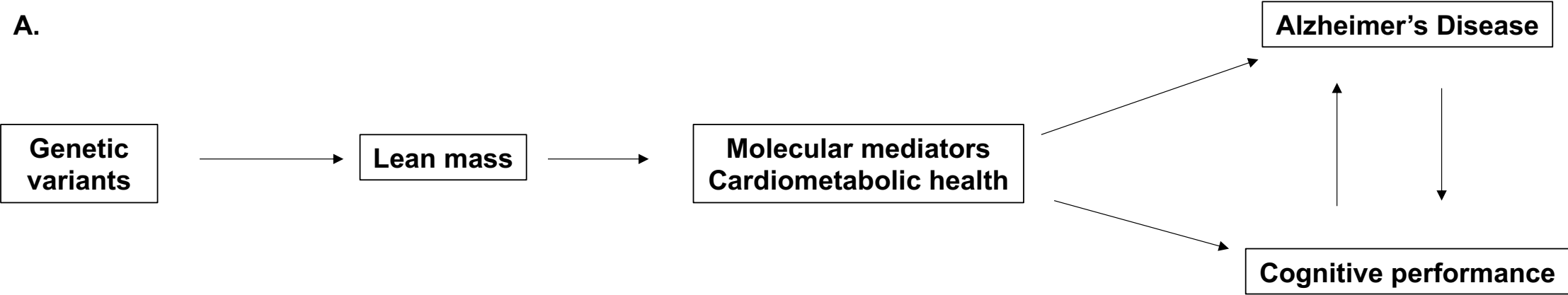

B.

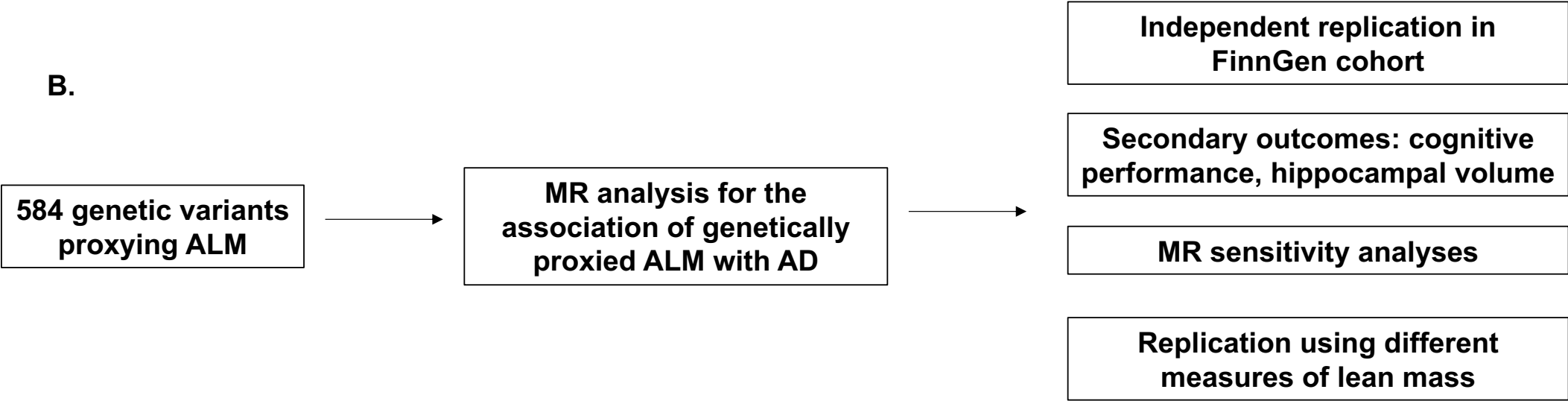

Supplement: Supplementary data [file bmjmed-2022-000354supp001.pdf]

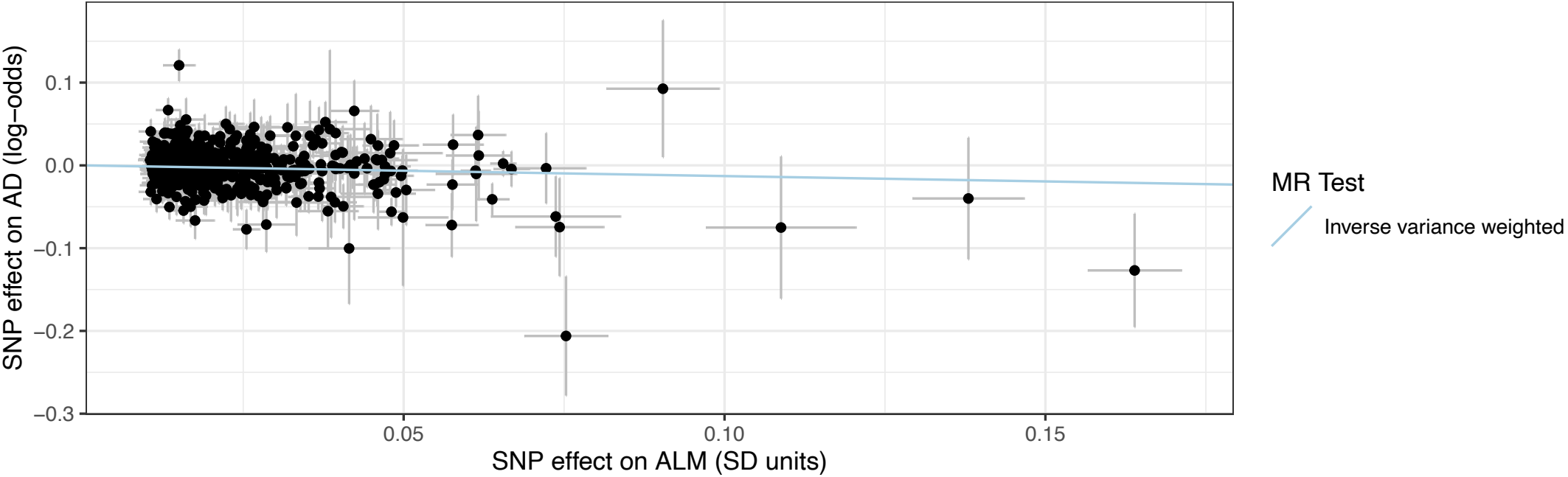

Supplement: Supplementary data [file bmjmed-2022-000354supp003.pdf]

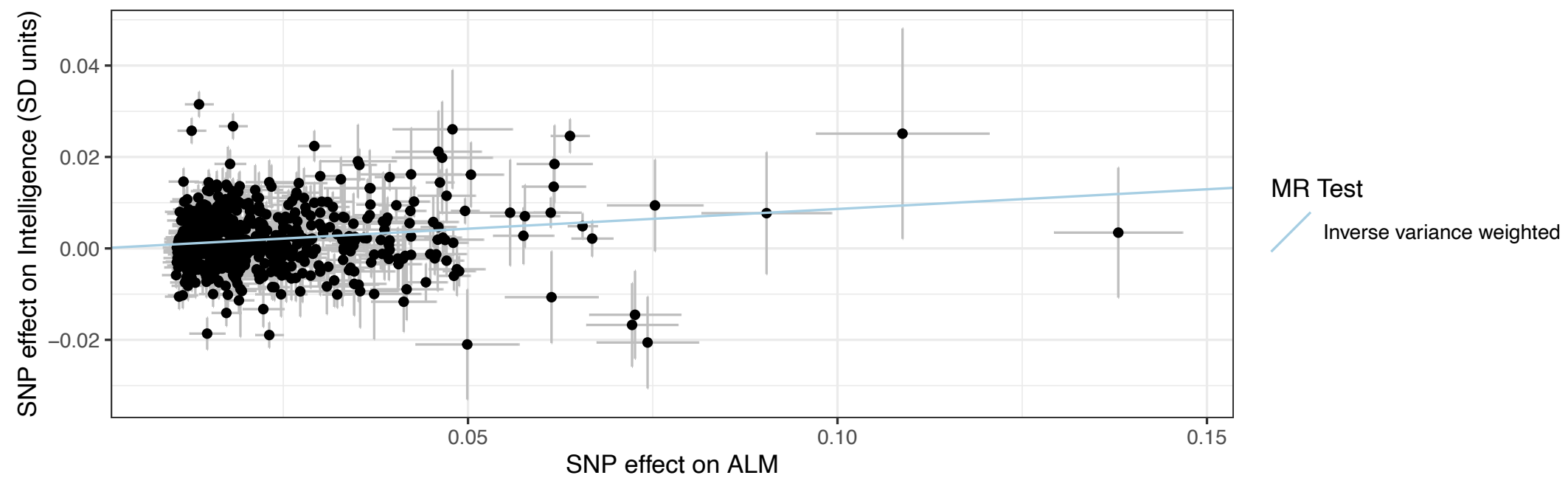

Supplement: Supplementary data [file bmjmed-2022-000354supp006.pdf]
