# Supplementary material for: Genetically proxied lean mass and risk of Alzheimer’s disease: mendelian randomisation study
Source: BMJ Med. 2023 Jun 29;2(1):e000354. doi: 10.1136/bmjmed-2022-000354 (PMC10410880; doi:10.1136/bmjmed-2022-000354)
Supplement: Supplementary data [file bmjmed-2022-000354supp002.pdf]

Genetic variants used in primary MR analysis to proxy appendicular lean mass adjusted for fat mass. SNP: single nucleotide polymorphism

SNP	Effect allele	Other allele	Beta	Standard err	P value
rs13170063	A	G	-0.0152	0.0019	4.11E-15
rs905938	T	C	-0.0394	0.0021	8.43E-77
rs7543136	T	C	-0.021	0.0021	9.96E-24
rs3001723	A	G	-0.0115	0.002	1.62E-08
rs73490624	T	C	-0.0184	0.0024	1.91E-14
rs6575589	A	G	-0.0117	0.002	3.36E-09
rs11243202	T	C	-0.0302	0.0019	2.83E-57
rs11210229	A	G	-0.0125	0.0019	8.21E-11
rs62228593	T	C	-0.0117	0.002	3.67E-09
rs35073631	T	C	-0.0112	0.0019	5.92E-09
rs7679276	A	G	0.033	0.0048	5.93E-12
rs34345560	A	G	0.0219	0.0024	7.10E-20
rs7082659	T	C	-0.0156	0.0028	2.27E-08
rs7418410	T	C	0.0155	0.0019	5.64E-16
rs7448554	A	C	-0.0132	0.002	1.77E-11
rs62515437	T	G	0.0369	0.0023	8.79E-60
rs261223	A	C	-0.0175	0.0019	2.30E-19
rs13321258	A	G	0.0133	0.0022	3.26E-09
rs7095087	A	G	0.0117	0.002	3.82E-09
rs60003758	A	G	0.0247	0.0025	1.71E-23
rs2115959	A	C	-0.0142	0.0019	5.70E-14
rs139921635	T	G	0.0385	0.0062	6.16E-10
rs57696574	A	C	-0.0173	0.002	4.50E-18
rs6693481	T	C	0.0143	0.002	2.21E-12
rs10832963	T	G	0.0203	0.0022	9.23E-21
rs73186333	A	C	-0.0373	0.0063	2.94E-09
rs9809116	A	G	0.016	0.0019	1.31E-16
rs2823990	A	G	-0.0127	0.002	4.19E-10
rs3769598	A	G	-0.0171	0.0027	1.32E-10
rs11633371	T	G	0.0216	0.0019	7.49E-30
rs113289555	T	G	-0.0206	0.0023	7.33E-20
rs6028716	A	G	-0.021	0.0022	4.58E-22
rs8018486	A	G	0.0138	0.0024	1.18E-08
rs310796	T	G	0.0142	0.002	2.53E-12
rs684905	T	C	-0.0118	0.0019	7.10E-10
rs28468602	T	G	-0.0112	0.0019	4.89E-09
rs2143407	A	G	0.0132	0.0021	2.06E-10
rs2754255	A	G	0.0153	0.0023	1.05E-11

rs9957318	A	G	-0.0187	0.002	1.02E-20
rs2236096	T	C	-0.018	0.0023	1.29E-15
rs2069408	A	G	0.0149	0.002	8.24E-14
rs71414738	T	C	0.015	0.0025	1.00E-09
rs76951439	T	C	0.0405	0.0042	6.49E-22
rs3792819	A	G	-0.021	0.0034	4.42E-10
rs7679806	A	G	0.0114	0.0021	4.59E-08
rs11221657	T	G	-0.0179	0.0028	1.21E-10
rs9634212	A	C	0.0471	0.0023	8.59E-95
rs2663126	A	G	-0.0139	0.0021	1.36E-11
rs8000973	T	C	-0.0134	0.0019	2.05E-12
rs395980	T	G	0.0184	0.0021	1.02E-17
rs4815952	T	C	0.0161	0.0019	1.24E-16
rs10922476	A	G	-0.016	0.0019	1.78E-17
rs7321635	A	C	0.0132	0.002	2.58E-11
rs591668	A	G	-0.0174	0.0019	2.00E-19
rs17197114	T	C	-0.0177	0.0025	1.54E-12
rs2296316	T	C	0.0192	0.0019	1.59E-23
rs13123591	T	G	-0.0185	0.002	2.35E-20
rs55681913	T	C	-0.0406	0.0031	2.11E-39
rs73052033	T	C	0.0151	0.0024	4.79E-10
rs12347137	A	C	0.046	0.0024	9.80E-85
rs10881582	A	G	-0.0151	0.0022	1.26E-11
rs3184504	T	C	-0.0183	0.0019	2.71E-22
rs61878760	A	G	0.019	0.0034	3.74E-08
rs2105333	T	G	0.019	0.002	1.70E-21
rs62048221	T	C	-0.0242	0.0022	4.92E-27
rs13209685	T	G	0.0277	0.0026	7.49E-27
rs7735891	T	C	0.0259	0.0019	1.14E-42
rs12907139	A	G	-0.0149	0.0019	4.89E-15
rs6000890	T	C	0.0135	0.002	8.17E-12
rs4395014	T	C	0.0131	0.0024	4.48E-08
rs1056747	A	G	0.0155	0.0019	8.05E-16
rs34287	A	G	0.0187	0.002	1.17E-20
rs6557894	T	C	-0.0123	0.0019	7.69E-11
rs57791149	T	C	0.0173	0.0019	3.26E-19
rs2071450	T	C	-0.0174	0.002	8.85E-19
rs718603	T	C	0.0131	0.0021	6.68E-10
rs9838614	T	G	0.0185	0.0019	1.21E-21
rs17718736	A	C	0.0115	0.002	1.39E-08
rs4380799	T	G	0.0255	0.0021	6.45E-33
rs10112506	A	G	0.012	0.0019	5.76E-10
rs67527161	T	C	0.0182	0.0023	5.79E-15

rs301805	T	G	0.0146	0.0019	1.66E-14
rs2584100	A	G	0.0108	0.0019	2.10E-08
rs80280630	T	C	-0.0168	0.003	2.28E-08
rs3782811	A	C	-0.0165	0.0022	3.96E-14
rs9388490	T	C	0.0462	0.0019	1.33E-130
rs72894003	T	C	-0.0423	0.0038	1.90E-28
rs4985445	A	G	0.0175	0.0019	3.30E-20
rs7610055	A	G	-0.0373	0.0029	3.55E-38
rs45474992	T	C	-0.0617	0.0051	2.17E-33
rs1530318	T	C	0.0209	0.0025	2.80E-17
rs42039	T	C	0.0481	0.0022	3.53E-106
rs10948	T	G	-0.0252	0.002	3.43E-36
rs7458576	A	G	0.0189	0.0019	7.13E-23
rs2005172	A	C	-0.048	0.002	2.35E-128
rs3782232	A	G	-0.0339	0.0037	2.41E-20
rs7320878	A	G	-0.015	0.0019	1.34E-14
rs12517711	T	C	0.0147	0.0019	2.79E-14
rs8019890	A	C	0.025	0.0019	1.96E-38
rs10107388	T	C	0.0159	0.002	6.95E-16
rs11060942	A	G	-0.0354	0.0052	7.49E-12
rs10202701	T	C	0.0227	0.0019	3.11E-33
rs532499	T	C	0.0127	0.0022	4.90E-09
rs3778858	T	G	0.0108	0.002	4.26E-08
rs1035583	A	G	0.0148	0.0019	1.99E-14
rs350895	T	C	0.0134	0.0021	3.50E-10
rs6910414	A	G	0.0142	0.0024	5.86E-09
rs3625	A	G	0.0148	0.0019	6.01E-15
rs62524625	T	C	0.0121	0.0021	5.21E-09
rs1514134	T	C	0.0114	0.0019	3.57E-09
rs9525326	A	G	0.0184	0.0024	3.62E-14
rs9361987	T	C	0.0115	0.0021	2.04E-08
rs112873218	T	C	0.0216	0.0031	4.02E-12
rs40270	A	C	-0.0151	0.0022	1.90E-11
rs680882	T	G	-0.0133	0.0022	1.98E-09
rs6874324	T	C	0.0132	0.0022	1.43E-09
rs12536902	A	C	0.0479	0.0081	3.66E-09
rs4752829	A	G	0.0262	0.0021	5.90E-36
rs1436164	T	C	-0.0139	0.0019	1.83E-13
rs12519407	A	C	-0.0181	0.0022	3.38E-17
rs28592876	A	G	0.03	0.0023	9.09E-38
rs951366	T	C	-0.0205	0.0019	9.15E-27
rs987666	A	G	0.0185	0.0029	2.33E-10
rs2602713	A	C	-0.0217	0.0019	1.68E-29

rs73108419	T	G	-0.0121	0.002	2.54E-09
rs2240735	T	C	0.0189	0.0022	3.99E-18
rs2101017	T	C	-0.0223	0.0028	1.44E-15
rs12351226	T	C	0.0218	0.0025	9.14E-18
rs61732778	A	G	0.023	0.0037	3.13E-10
rs2347808	A	G	-0.0125	0.0019	5.79E-11
rs36048468	T	C	0.0254	0.0023	9.34E-28
rs62143873	A	G	-0.0115	0.0019	1.19E-09
rs111622870	T	C	0.0282	0.0044	1.86E-10
rs73856768	T	C	0.0247	0.0035	1.55E-12
rs1584011	T	G	-0.0159	0.002	9.78E-16
rs62370472	T	C	0.0253	0.0023	1.45E-27
rs4938359	A	G	0.0156	0.0024	3.24E-11
rs173135	T	C	-0.0341	0.003	3.25E-30
rs4683435	A	G	-0.0144	0.0022	1.61E-10
rs2324154	A	C	0.015	0.0019	1.92E-15
rs7522400	A	G	-0.0129	0.0022	5.63E-09
rs190823861	A	G	-0.0345	0.0045	2.09E-14
rs10975935	A	G	0.0121	0.0022	4.15E-08
rs249677	A	C	-0.0109	0.002	2.40E-08
rs10807137	T	C	-0.0455	0.0025	5.68E-75
rs13430869	T	G	0.0272	0.0021	6.37E-37
rs60389750	T	C	-0.0175	0.0021	1.06E-16
rs6675858	T	C	-0.0137	0.0023	2.40E-09
rs28529426	T	C	-0.0168	0.0026	5.82E-11
rs17400325	T	C	-0.0345	0.0047	2.10E-13
rs10993916	T	C	0.0148	0.0021	7.80E-13
rs4077103	A	C	-0.0143	0.0026	4.18E-08
rs9394169	A	G	-0.0178	0.0019	6.95E-21
rs72771070	T	C	0.015	0.0021	1.30E-12
rs7731023	A	G	-0.0166	0.0019	3.48E-18
rs62033029	A	G	-0.0141	0.0023	1.73E-09
rs7231886	A	G	-0.0106	0.0019	3.44E-08
rs6582398	T	C	0.014	0.002	1.14E-12
rs7485647	A	G	-0.0261	0.0026	1.05E-23
rs1430157	T	C	0.0182	0.002	3.24E-19
rs76895963	T	G	-0.1639	0.0073	8.23E-112
rs9483209	T	C	0.0182	0.0026	2.40E-12
rs10864899	A	G	0.0112	0.0019	3.85E-09
rs35756741	T	C	-0.0378	0.0033	5.80E-31
rs7816345	T	C	0.0255	0.0025	6.23E-24
rs292168	A	G	0.0282	0.0019	8.33E-50
rs10514518	T	C	0.0171	0.002	8.28E-17

rs12423821	T	C	-0.0161	0.0027	1.17E-09
rs7910211	T	C	-0.0175	0.0026	1.52E-11
rs9905385	A	G	0.0339	0.002	1.94E-63
rs9594714	T	G	0.0144	0.0021	2.65E-12
rs76517946	A	C	-0.0368	0.0035	1.69E-26
rs9517483	A	G	0.0181	0.0021	2.26E-18
rs3116602	T	G	0.0612	0.0023	9.52E-155
rs12943867	A	G	0.0184	0.002	7.57E-20
rs55758152	A	G	0.0145	0.002	1.05E-12
rs11588796	T	C	0.0289	0.0052	2.97E-08
rs72809820	T	C	-0.0111	0.002	3.13E-08
rs4788218	T	C	-0.0275	0.0019	5.53E-46
rs3764002	T	C	0.028	0.0021	4.47E-39
rs117203652	A	G	-0.0346	0.0055	4.32E-10
rs6082354	A	C	0.024	0.002	1.19E-32
rs2283200	T	C	-0.0281	0.0042	1.48E-11
rs34776209	T	C	-0.0317	0.0022	1.78E-47
rs73006226	A	C	-0.0182	0.0029	2.23E-10
rs7701233	T	C	0.0179	0.0019	5.12E-21
rs58646822	T	C	-0.0128	0.0022	5.01E-09
rs62113892	T	C	-0.0155	0.0025	3.68E-10
rs1285080	A	C	0.0234	0.0034	2.80E-12
rs4735761	A	C	-0.0331	0.0021	3.66E-56
rs2788213	A	G	0.0123	0.0021	3.80E-09
rs2142644	A	C	-0.0181	0.002	3.37E-19
rs757834	T	C	-0.0256	0.0024	1.25E-25
rs9521733	T	C	-0.0167	0.002	1.88E-17
rs12051245	T	C	-0.0299	0.0022	2.56E-40
rs6060355	A	G	0.0655	0.002	1.00E-200
rs10202845	A	G	0.0288	0.003	5.35E-22
rs11198591	A	G	0.0148	0.002	4.91E-14
rs35268848	A	C	0.0737	0.0101	2.83E-13
rs73114154	T	C	0.0138	0.002	1.73E-12
rs2923411	T	C	-0.0127	0.0019	4.77E-11
rs577961	T	C	0.0135	0.0019	7.50E-13
rs6852065	T	C	0.0131	0.0019	6.63E-12
rs10796828	T	G	-0.0154	0.002	5.77E-15
rs116339650	A	G	0.0175	0.0029	1.05E-09
rs10776560	T	C	-0.0157	0.0019	7.88E-17
rs28485212	T	C	-0.0188	0.0027	1.24E-12
rs2209098	T	C	-0.024	0.002	1.73E-32
rs7858712	A	G	-0.0347	0.0034	1.04E-24
rs12894822	A	G	-0.0135	0.0022	5.50E-10

rs2539251	T	G	-0.0162	0.0027	2.62E-09
rs11721522	A	G	-0.0106	0.0019	4.03E-08
rs4852257	T	G	0.0231	0.0019	6.21E-34
rs74494415	T	C	-0.0417	0.0049	1.82E-17
rs4121583	T	C	0.0118	0.002	4.62E-09
rs57513571	T	C	-0.0191	0.0024	7.02E-16
rs80295797	T	C	-0.0198	0.002	3.83E-23
rs545104	T	C	-0.0127	0.002	8.08E-11
rs1260326	T	C	-0.0323	0.0019	6.16E-64
rs4979576	T	C	0.0317	0.0029	5.61E-27
rs2303423	T	C	-0.0168	0.003	2.64E-08
rs12907384	T	C	0.0269	0.0019	1.60E-45
rs61940146	A	G	-0.0112	0.002	1.27E-08
rs57481308	T	C	0.0114	0.002	5.99E-09
rs2678898	T	C	0.0129	0.0019	3.09E-11
rs2521349	A	G	0.0155	0.0019	2.01E-15
rs7220127	T	C	0.0105	0.0019	4.60E-08
rs55745410	A	G	0.0157	0.002	1.59E-15
rs6543146	T	G	-0.0154	0.0019	4.19E-16
rs8017006	A	G	-0.0122	0.002	2.22E-09
rs9364192	A	G	-0.0142	0.0019	1.19E-13
rs41271299	T	C	0.0616	0.0043	3.19E-47
rs10764692	A	C	0.0111	0.002	1.62E-08
rs11206749	A	G	-0.0304	0.0024	9.88E-38
rs143554698	T	C	-0.0257	0.0027	3.39E-21
rs56136528	T	G	0.0243	0.0043	2.26E-08
rs9636364	A	G	0.011	0.0019	5.09E-09
rs34390533	A	C	-0.0257	0.0022	6.69E-32
rs7902	A	G	-0.0149	0.0019	5.22E-15
rs28379706	T	C	-0.0114	0.002	4.49E-09
rs6762851	T	C	0.0218	0.002	1.50E-28
rs112153300	A	G	0.0261	0.0034	7.05E-15
rs34522021	T	C	0.0126	0.0019	3.38E-11
rs11612462	T	G	-0.015	0.0025	2.55E-09
rs7768382	T	C	0.0201	0.0019	1.57E-26
rs1527067	T	G	0.0122	0.002	4.19E-10
rs2592208	A	C	-0.0124	0.0019	5.52E-11
rs720508	A	G	-0.0172	0.0031	1.71E-08
rs35696197	T	G	0.0127	0.0021	3.94E-09
rs11098677	T	G	-0.0263	0.0023	3.94E-30
rs72841270	T	G	-0.0294	0.0028	2.25E-26
rs8887	T	C	0.0167	0.0019	5.37E-18
rs4602848	A	G	-0.016	0.002	3.15E-15

rs73384223	T	C	0.0205	0.0024	1.27E-17
rs10203320	T	C	-0.0138	0.002	7.86E-12
rs12099669	A	G	0.0331	0.002	1.39E-58
rs73833889	T	G	-0.0127	0.0021	2.78E-09
rs112021215	T	C	0.0147	0.0025	5.99E-09
rs111365325	T	C	-0.0271	0.0022	1.12E-33
rs4847378	T	G	0.0136	0.0019	1.64E-12
rs13391980	A	G	-0.0225	0.0029	7.60E-15
rs10845408	T	C	0.0255	0.002	3.25E-38
rs6570509	T	G	-0.0244	0.0021	1.27E-31
rs11672848	T	C	-0.0171	0.0019	7.73E-19
rs4965298	T	C	-0.0119	0.0021	1.81E-08
rs6931421	T	G	0.0279	0.002	2.31E-43
rs7247738	A	G	0.0146	0.002	3.63E-13
rs6874142	T	G	-0.0288	0.0031	5.15E-20
rs3818416	A	C	-0.0279	0.0022	2.01E-35
rs4504126	A	C	-0.046	0.0058	1.62E-15
rs12962050	A	G	0.0153	0.002	1.52E-14
rs7359097	T	C	0.0121	0.0019	5.51E-10
rs6977416	A	G	0.0457	0.002	1.43E-113
rs78766798	T	C	-0.0319	0.0035	2.61E-20
rs2112617	A	G	-0.0167	0.0019	1.07E-18
rs13127468	A	C	-0.0123	0.0019	9.86E-11
rs75702986	A	G	-0.0163	0.0025	3.14E-11
rs10176654	T	C	0.0128	0.0023	1.70E-08
rs743577	A	G	-0.0237	0.0021	1.41E-28
rs62501195	A	C	0.0198	0.0025	8.11E-15
rs1014526	T	C	0.0138	0.002	1.87E-12
rs76364830	A	G	-0.0471	0.0039	2.70E-33
rs4976262	T	C	0.0245	0.002	4.37E-33
rs9426827	T	C	0.0109	0.0019	5.33E-09
rs75022676	A	G	-0.0163	0.0023	2.84E-12
rs12997625	T	C	-0.017	0.0019	1.50E-19
rs496783	A	G	0.0124	0.0019	8.13E-11
rs949214	A	C	0.019	0.0024	2.71E-15
rs704660	T	C	0.0153	0.0019	2.28E-15
rs9590328	A	G	-0.0153	0.0027	2.02E-08
rs900399	A	G	-0.0164	0.0019	1.35E-17
rs8136517	T	C	-0.0267	0.0039	6.58E-12
rs12904298	A	G	0.0152	0.0023	4.49E-11
rs10205141	A	G	-0.0241	0.0044	4.71E-08
rs9344126	T	C	0.0185	0.0019	2.21E-22
rs115912456	A	G	-0.0577	0.0047	3.69E-34

rs2789365	T	C	-0.0145	0.0019	1.14E-14
rs72740637	A	G	0.0219	0.0021	4.91E-25
rs4799799	A	C	-0.0113	0.002	1.29E-08
rs76520574	T	C	-0.0449	0.0049	2.09E-20
rs12655296	T	C	-0.011	0.002	1.62E-08
rs12212816	T	C	0.0162	0.0019	8.26E-18
rs9391254	T	C	0.0166	0.002	2.10E-16
rs2236406	T	C	-0.0394	0.002	1.26E-87
rs6544743	T	G	0.0217	0.0024	1.06E-19
rs72656010	T	C	0.0668	0.0028	7.31E-126
rs72753091	T	C	-0.0333	0.0055	1.99E-09
rs59985551	T	C	-0.0313	0.0022	2.43E-44
rs11720869	A	G	0.0141	0.002	2.54E-12
rs9894577	A	G	-0.031	0.002	1.40E-52
rs4911377	A	G	0.0194	0.0019	3.19E-24
rs7570235	T	C	0.0168	0.0019	2.08E-18
rs3103223	T	C	-0.0126	0.0022	6.02E-09
rs12533452	T	C	0.0237	0.0026	1.31E-19
rs7633464	A	G	0.0175	0.0019	1.28E-20
rs12483401	T	C	0.0387	0.0067	9.22E-09
rs10019221	T	G	-0.0124	0.0019	1.22E-10
rs67551338	T	C	0.0576	0.004	1.04E-47
rs4655345	A	G	0.0246	0.0019	5.81E-38
rs4682483	A	G	-0.0165	0.0026	2.65E-10
rs11605297	A	G	0.0146	0.0022	8.03E-11
rs10128781	T	C	-0.0172	0.0021	1.69E-16
rs1345203	T	C	-0.0192	0.0023	7.48E-17
rs33973388	T	G	0.0249	0.0019	1.45E-38
rs10119967	A	C	-0.0353	0.0023	4.43E-51
rs8054549	A	C	-0.0251	0.0019	3.37E-39
rs2229840	T	C	0.0341	0.0026	3.02E-40
rs117068593	T	C	0.0403	0.0024	8.83E-62
rs1168768	T	C	0.0332	0.006	3.61E-08
rs10749157	T	C	-0.0113	0.002	1.24E-08
rs13177933	A	G	-0.0153	0.0019	2.98E-15
rs2305141	A	G	-0.0183	0.0019	1.08E-21
rs3115084	T	C	0.024	0.0031	5.34E-15
rs2925155	T	C	-0.015	0.0022	5.47E-12
rs11651280	T	G	0.0267	0.004	1.79E-11
rs112537273	T	C	0.0212	0.0022	3.34E-21
rs11191208	A	G	0.0147	0.0024	3.69E-10
rs4622329	A	G	0.0149	0.002	8.62E-14
rs11158820	A	G	0.0235	0.0021	5.50E-30

rs11684531	A	G	0.0172	0.0028	4.17E-10
rs7974773	A	G	0.0121	0.0022	1.97E-08
rs60408354	A	G	0.0259	0.0036	1.15E-12
rs6962887	T	G	0.0127	0.0021	6.20E-10
rs35811052	A	G	0.0148	0.0022	8.84E-12
rs2993531	A	C	-0.0176	0.0019	1.74E-20
rs55633823	T	C	0.0146	0.0022	3.28E-11
rs9832919	A	G	0.0179	0.002	7.90E-20
rs12074850	A	G	-0.0393	0.0033	2.72E-33
rs9659073	A	G	-0.0198	0.0019	5.05E-26
rs55872725	T	C	0.0222	0.0019	1.46E-30
rs112369231	T	C	-0.0181	0.0021	2.63E-17
rs7253742	T	C	0.0167	0.0019	5.66E-18
rs1472852	A	C	-0.0638	0.0026	8.22E-135
rs11880992	A	G	0.0245	0.0019	6.70E-37
rs36012032	A	C	0.0298	0.0033	9.93E-20
rs2212926	A	C	-0.022	0.0023	7.75E-21
rs1063582	T	G	0.0185	0.0022	1.12E-16
rs6470771	A	C	0.0268	0.0025	1.55E-26
rs332116	T	C	-0.0206	0.0021	2.89E-22
rs7107356	A	G	-0.0133	0.0019	1.86E-12
rs7185244	T	C	0.0148	0.0023	1.40E-10
rs2871960	A	C	-0.0469	0.0019	2.17E-135
rs11217863	A	G	-0.0268	0.003	1.06E-19
rs7730092	T	G	0.0131	0.0024	4.81E-08
rs4073154	A	G	-0.0274	0.0023	1.93E-33
rs6721191	A	G	0.0144	0.0019	3.17E-14
rs36000545	A	G	0.022	0.002	2.56E-29
rs7016759	T	C	-0.0231	0.0025	8.50E-20
rs7952436	T	C	-0.0453	0.0034	1.62E-39
rs5004102	A	C	-0.0153	0.0019	9.87E-16
rs7654502	T	C	0.0134	0.002	1.44E-11
rs1443536	A	G	-0.0218	0.0021	1.91E-26
rs655113	T	C	-0.0188	0.0021	7.42E-20
rs144109601	A	C	-0.0278	0.0048	5.15E-09
rs702886	A	G	-0.012	0.002	1.11E-09
rs568267	T	C	0.0122	0.0022	2.23E-08
rs11042717	T	C	0.029	0.0019	4.04E-53
rs35624335	T	C	-0.0134	0.0021	2.29E-10
rs670318	T	C	-0.0413	0.0044	2.52E-21
rs72657800	T	C	0.0219	0.0035	6.39E-10
rs28529055	T	G	-0.0147	0.0019	1.88E-14
rs35816944	A	G	-0.1088	0.0117	1.27E-20

rs12047986	A	G	0.0126	0.0019	1.82E-11
rs17713523	A	G	0.0114	0.0019	3.05E-09
rs6054390	A	G	-0.0188	0.002	1.45E-21
rs2965076	A	G	-0.0151	0.0024	1.70E-10
rs2019203	A	C	0.0189	0.0019	1.84E-23
rs12340775	A	G	-0.0287	0.0043	1.73E-11
rs9266244	A	G	-0.0427	0.0021	1.21E-94
rs3822742	A	C	0.0162	0.002	1.03E-16
rs78378222	T	G	-0.138	0.0087	4.51E-56
rs4802635	A	G	0.0117	0.0019	1.89E-09
rs17478946	A	G	0.0192	0.0021	9.98E-21
rs72829852	T	C	0.0309	0.0039	3.74E-15
rs55852614	T	C	0.0393	0.0022	3.29E-73
rs2230033	A	G	-0.0265	0.0019	3.49E-43
rs336630	T	C	-0.0106	0.0019	2.90E-08
rs17278379	T	C	-0.0226	0.0029	2.40E-15
rs12595051	A	G	0.0179	0.0021	2.84E-17
rs61775902	T	C	-0.0186	0.002	1.74E-21
rs246177	T	C	0.0214	0.002	2.04E-27
rs1325596	A	G	0.0287	0.0019	2.77E-52
rs45528934	T	C	0.0262	0.0026	1.97E-24
rs7828086	T	C	-0.0135	0.0022	1.11E-09
rs31196	A	C	-0.0107	0.0019	2.07E-08
rs10822117	A	G	0.0176	0.0022	4.24E-15
rs12512942	A	G	-0.0162	0.002	1.58E-16
rs78457529	T	C	-0.0904	0.0088	1.22E-24
rs56017587	T	C	-0.0156	0.0028	1.38E-08
rs113827862	T	C	0.0235	0.004	4.67E-09
rs28736838	T	C	-0.0117	0.002	1.07E-08
rs17066856	T	C	0.0185	0.0033	2.10E-08
rs55922628	A	C	-0.0201	0.002	1.25E-24
rs1047891	A	C	0.0233	0.002	5.70E-31
rs488621	A	G	-0.0191	0.0019	2.86E-24
rs56207600	A	G	0.0192	0.003	2.50E-10
rs10748128	T	G	0.0255	0.002	6.77E-38
rs2885697	T	G	-0.0323	0.002	9.21E-60
rs12188208	A	C	0.0195	0.0022	1.40E-18
rs1556659	T	C	0.0163	0.002	7.19E-17
rs3828729	A	G	0.016	0.002	4.67E-15
rs14976	T	C	0.0144	0.002	1.43E-12
rs2194411	A	G	0.0443	0.0029	2.43E-54
rs861598	A	C	-0.0129	0.0019	8.15E-12
rs7095472	A	G	-0.0267	0.0019	7.66E-45

rs1899040	T	C	0.0152	0.0023	9.04E-11
rs2287821	T	C	-0.0153	0.0019	6.95E-16
rs7014590	T	C	0.0228	0.0022	4.48E-26
rs2223966	A	G	0.0186	0.0022	5.63E-17
rs116008080	A	G	-0.0415	0.0063	4.06E-11
rs1730028	T	G	-0.0131	0.0019	7.39E-12
rs723149	A	G	0.0276	0.0019	1.43E-47
rs143076454	A	G	-0.0499	0.007	1.06E-12
rs4886708	T	C	0.0188	0.0022	4.87E-18
rs59950280	A	G	-0.0254	0.002	7.32E-36
rs66922415	A	G	-0.0488	0.0022	1.10E-104
rs17205463	T	C	-0.0263	0.0019	4.21E-43
rs35963161	A	G	-0.0157	0.0019	7.46E-16
rs2035901	A	G	-0.024	0.0019	9.43E-37
rs2142331	T	C	-0.0165	0.0019	1.38E-17
rs2570515	T	C	-0.0181	0.0022	4.05E-17
rs17681189	A	C	-0.0131	0.0019	5.82E-12
rs331917	A	G	0.0127	0.0019	3.54E-11
rs41311445	A	C	0.0328	0.0032	4.73E-24
rs35732917	T	C	-0.0204	0.0021	2.08E-22
rs1005723	T	C	0.0161	0.0024	1.79E-11
rs2676298	T	C	-0.0269	0.0027	2.39E-23
rs7563362	A	G	-0.0352	0.0027	3.27E-39
rs2651472	T	G	0.0108	0.0019	1.22E-08
rs291979	A	G	0.0242	0.0023	6.76E-27
rs4252548	T	C	-0.0753	0.0065	2.96E-31
rs12541381	A	G	-0.0319	0.0022	2.81E-49
rs9669278	T	C	0.0496	0.0019	5.25E-151
rs7328187	T	G	-0.0116	0.0019	1.19E-09
rs12616192	A	G	-0.0261	0.0038	6.83E-12
rs17246129	A	G	0.0254	0.002	1.27E-35
rs4274112	A	G	0.0217	0.002	2.47E-28
rs68049170	A	G	-0.0259	0.0021	2.69E-34
rs2607234	A	G	0.0302	0.0043	2.00E-12
rs876122	A	G	-0.0162	0.0029	2.19E-08
rs2181834	T	G	0.0254	0.0019	7.70E-41
rs28678024	A	G	0.0119	0.0021	1.90E-08
rs17773965	T	C	-0.0163	0.0027	1.51E-09
rs1317349	T	G	-0.0258	0.0021	6.89E-35
rs116092985	A	G	0.0401	0.0033	1.17E-34
rs117818446	A	G	0.0423	0.0068	5.29E-10
rs79592437	T	C	0.0205	0.0029	7.87E-13
rs13109280	A	G	-0.0131	0.002	9.15E-11

rs7367519	T	C	-0.0164	0.002	4.68E-16
rs35288270	T	C	0.0328	0.0028	3.44E-32
rs10068640	A	G	0.0112	0.002	1.28E-08
rs3814333	T	C	0.0182	0.002	3.26E-19
rs2029134	T	G	0.0126	0.002	2.00E-10
rs6593210	A	G	0.0146	0.0024	4.88E-10
rs10123619	A	G	0.0171	0.0026	4.05E-11
rs28757154	A	G	-0.0193	0.0027	1.90E-12
rs1004982	T	C	0.0117	0.002	1.92E-09
rs713467	A	G	0.0146	0.0019	3.09E-14
rs1468177	T	C	-0.0181	0.002	7.07E-20
rs1444628	T	C	0.024	0.002	6.85E-32
rs10453441	A	G	0.0139	0.002	9.10E-13
rs738086	T	G	0.0214	0.0024	9.53E-19
rs12702693	T	C	0.0173	0.0019	6.56E-20
rs9828525	T	C	0.0121	0.0019	2.51E-10
rs2274351	T	C	0.017	0.0019	3.07E-19
rs1341215	A	G	0.0229	0.0027	6.32E-17
rs61729527	T	C	-0.0346	0.0043	4.86E-16
rs10019880	A	G	-0.0189	0.0032	3.15E-09
rs7229520	A	G	-0.0224	0.002	9.21E-29
rs77442664	T	C	-0.0286	0.0045	2.90E-10
rs4763327	T	C	0.0123	0.0021	2.04E-09
rs10483727	T	C	0.0368	0.0019	6.73E-80
rs2748501	A	G	-0.0195	0.0019	1.31E-24
rs73125634	T	G	-0.0195	0.0021	5.11E-20
rs1797070	A	G	0.0219	0.0021	4.93E-25
rs4836544	T	C	0.0175	0.003	4.26E-09
rs9696477	T	C	-0.0154	0.0023	5.29E-11
rs7598430	T	C	-0.016	0.0019	1.37E-17
rs2454390	T	C	0.0176	0.0026	1.74E-11
rs73413540	T	C	-0.0124	0.0023	4.42E-08
rs116493405	A	G	0.0287	0.0042	9.52E-12
rs6844176	T	C	-0.0129	0.0019	1.21E-11
rs10242866	T	C	0.0157	0.0019	3.66E-16
rs56239180	T	G	0.0459	0.0062	1.85E-13
rs12713004	A	G	-0.0367	0.0021	2.40E-68
rs9375188	T	C	0.0136	0.0019	6.80E-13
rs61944841	A	G	0.0253	0.002	3.54E-37
rs7020491	T	C	-0.0178	0.0019	1.22E-20
rs244711	T	C	0.0279	0.0022	1.54E-37
rs13209574	T	G	-0.0292	0.0032	2.64E-20
rs604723	T	C	0.0166	0.0021	8.16E-15

rs990315	T	C	0.0115	0.002	5.07E-09
rs6505216	T	G	-0.0498	0.0023	1.83E-101
rs2569888	A	G	0.0133	0.0022	2.29E-09
rs62103240	A	G	0.0212	0.0037	1.40E-08
rs1405227	A	G	0.0129	0.002	1.57E-10
rs36226649	T	C	-0.0485	0.0038	3.05E-37
rs2764264	T	C	0.0203	0.0021	5.07E-23
rs60804050	A	G	-0.0217	0.0021	5.01E-24
rs963317	A	G	0.0136	0.002	1.03E-11
rs10283100	A	G	-0.0575	0.0041	4.11E-44
rs798548	T	C	0.0359	0.0021	2.86E-68
rs34517439	A	C	0.0421	0.0029	5.80E-48
rs80132799	T	C	0.0231	0.0038	1.31E-09
rs12344515	T	C	-0.0163	0.0022	2.28E-13
rs62621812	A	G	0.0743	0.0069	3.16E-27
rs10075249	T	C	0.0143	0.0019	4.56E-14
rs2140619	A	G	-0.0113	0.0019	5.18E-09
rs77809369	T	C	0.0237	0.0039	9.84E-10
rs7129320	A	G	-0.0389	0.0025	7.29E-53
rs2627692	T	C	-0.0177	0.0019	7.24E-21
rs926436	A	G	0.0178	0.0027	3.84E-11
rs12700901	A	C	-0.0184	0.0019	1.67E-21
rs2237485	A	G	0.0191	0.0023	3.73E-17
rs8084413	A	G	-0.0127	0.0019	3.25E-11
rs62106258	T	C	0.0504	0.0044	6.45E-31
rs10225945	A	G	0.0146	0.0026	3.28E-08
rs10815274	A	C	-0.0124	0.0019	6.46E-11
rs6738207	A	G	0.0127	0.0019	4.15E-11
rs147110934	T	G	-0.0722	0.0062	9.39E-32
rs2070598	A	G	0.0204	0.0019	6.36E-27
rs11187838	A	G	0.0394	0.0019	1.16E-94
rs7893378	A	G	0.0175	0.0031	2.36E-08
rs113232639	A	G	0.0327	0.0019	4.79E-64
rs11014285	A	G	0.0342	0.0026	2.89E-40
rs6558167	T	C	0.0115	0.002	6.14E-09
rs700677	A	C	0.0173	0.002	1.13E-18
rs165849	A	G	0.0157	0.0021	4.57E-14
rs7301341	T	C	0.0255	0.002	9.27E-37
rs6849302	A	G	-0.0155	0.0024	7.11E-11
rs377599	T	C	0.0217	0.0019	3.50E-29
rs4282339	A	G	-0.0311	0.0023	6.16E-41
rs4244809	A	G	-0.0262	0.0023	5.55E-29
rs543650	T	G	-0.025	0.002	1.49E-37

rs6821305	A	C	-0.0204	0.0019	3.15E-26
rs56363908	A	G	0.0382	0.0047	3.88E-16
rs5742915	T	C	-0.0248	0.0019	9.33E-39
rs2138374	T	C	0.0149	0.002	2.79E-13
rs4748008	T	C	0.0125	0.0019	8.95E-11
rs1823217	A	G	0.0181	0.002	4.06E-20
rs6502935	T	C	-0.0125	0.0022	6.32E-09
rs7689420	T	C	-0.0466	0.0025	1.50E-76
rs13205819	A	G	-0.0213	0.0026	2.40E-16
rs10128333	T	C	-0.0146	0.0025	9.51E-09
rs781669	T	C	0.0164	0.0019	3.14E-18
rs34879158	A	C	0.0363	0.0022	1.55E-63
rs74379684	T	C	-0.0272	0.0036	4.39E-14
rs13037813	T	C	-0.0292	0.0022	1.71E-39
rs2390669	A	C	-0.0174	0.0028	7.00E-10
rs200439	A	C	0.0128	0.0023	1.51E-08
rs3768495	T	C	-0.0178	0.0021	1.07E-17
rs8904	A	G	-0.0157	0.002	1.52E-15
rs14044009	A	G	0.0613	0.0063	1.45E-22
rs62466110	T	C	0.0371	0.0041	5.74E-20
rs1786263	T	G	-0.019	0.0019	1.03E-22
rs7906411	T	C	0.0143	0.0022	3.23E-11
rs4752689	A	G	0.0205	0.0019	1.36E-26
rs212526	T	C	-0.0214	0.0019	3.84E-29
rs11367110	T	C	0.015	0.0023	4.23E-11
rs61781373	A	C	-0.0159	0.0024	7.75E-11
rs144627572	A	G	0.0439	0.0053	1.30E-16
rs447352	T	C	-0.0181	0.0029	6.62E-10
rs2578565	T	C	-0.0141	0.002	1.37E-12
rs11629979	A	G	0.0196	0.0021	3.17E-20
