# Supplementary material for: Genetically proxied lean mass and risk of Alzheimer’s disease: mendelian randomisation study
Source: BMJ Med. 2023 Jun 29;2(1):e000354. doi: 10.1136/bmjmed-2022-000354 (PMC10410880; doi:10.1136/bmjmed-2022-000354)
Supplement: Supplementary data [file bmjmed-2022-000354supp004.pdf]

Supplementary Table 3. Multivariable Mendelian randomization estimates for the association of genetically proxied appendicular lean mass (adjusted for fat mass) with AD after adjustment for genetic predictors of height. Results from fixed-effects meta-analysis are displayed in the last row. CI: confidence interval; IVW: inverse-variance weighted.

**Cohort**	Beta [95% CI]	*P* value
UKB (682 total SNPs)	0.91 [0.80-1.05]	0.19
FinnGen (679 total SNPs)	0.86 [0.72-1.03]	0.11
Meta-analysis	0.90 [0.80-0.997]	0.04
