# Supplementary material for: Genetically proxied lean mass and risk of Alzheimer’s disease: mendelian randomisation study
Source: BMJ Med. 2023 Jun 29;2(1):e000354. doi: 10.1136/bmjmed-2022-000354 (PMC10410880; doi:10.1136/bmjmed-2022-000354)
Supplement: Supplementary data [file bmjmed-2022-000354supp005.pdf]

Supplementary Table 2. Mendelian randomization estimates for the association of genetically proxied liability to AD with appendicular lean mass adjusted for fat mass. 16 SNPs were used in MR analysis. CI: confidence interval; IVW: inverse-variance weighted.

**Method**	Beta [95% CI]	*P* value
IVW	0.01 [0.001-0.02]	0.03
Weighted Median	0.01 [0.004-0.02]	0.002
MR Egger	0.01 [-0.005-0.02]	0.28
Penalised weighted median	0.01 [0.005-0.02]	0.001
